# A Pilot Study Investigating the Dynamics of Pigeon Circovirus Recombination in Domesticated Pigeons Housed in a Single Loft

**DOI:** 10.3390/v13060964

**Published:** 2021-05-22

**Authors:** Anthony Khalifeh, Simona Kraberger, Daria Dziewulska, Arvind Varsani, Tomasz Stenzel

**Affiliations:** 1 Biodesign Center for Fundamental and Applied Microbiomics, Center for Evolution and Medicine, School of Life Sciences, Arizona State University, Tempe, AZ 85287, USA; akhalif5@asu.edu (A.K.); simona.kraberger@asu.edu (S.K.); 2 Department of Poultry Diseases, Faculty of Veterinary Medicine, University of Warmia and Mazury, 10-719 Olsztyn, Poland; daria.pestka@uwm.edu.pl; 3 Structural Biology Research Unit, Department of Integrative Biomedical Sciences, University of Cape Town, Observatory, Cape Town 7701, South Africa

**Keywords:** evolution, genome, one loft, pigeons, pigeon circovirus, recombination

## Abstract

Pigeon circovirus (PiCV) infects pigeon populations worldwide and has been associated with immunosuppression in younger pigeons. Recombination is a common mechanism of evolution that has previously been shown in various members of the *Circoviridae* family, including PiCV. In this study, three groups of pigeons acquired from separate lofts were screened for PiCV, and their genome sequence was determined. Following this, they were housed in a single loft for 22 days, during which blood and cloacal swab samples were taken. From these blood and cloacal swabs, PiCV genomes were determined with the aim to study the spread and recombination dynamics of PiCV in the birds. Genome sequences of PiCV were determined from seven pigeons (seven tested PiCV positive) before they were housed together in a loft (*n* = 58 sequences) and thereafter from the ten pigeons from blood and cloacal swabs (*n* = 120). These 178 PiCV genome sequences represent seven genotypes (98% pairwise identity genotype demarcation), and they share >88% genome-wide pairwise identity. Recombination analysis revealed 13 recombination events, and a recombination hotspot spanning the 3′ prime region, the replication-associated protein (*rep*) gene and the intergenic region. A cold spot in the capsid protein-coding region of the genome was also identified. The majority of the recombinant regions were identified in the *rep* coding region. This study provides insights into the evolutionary dynamics of PiCV in pigeons kept under closed rearing systems.

## 1. Introduction

The domestic pigeon (*Columba livia f. domestica*), a species in the Columbidae family, is strongly associated with larger human populations [1]. Wild ancestors of domestic pigeons—the rock pigeon (*Columba livia*) can flock in groups of up to 400 individuals, and their range can span several kilometers. Similar behavior of living in groups is also observed in the feral domestic pigeon population inhabiting cities around the world, which are referred to as urban pigeons [2]. Nowadays, numerous pigeon breeds are known, and some are used for either meat production, such as ornamental birds, or for pigeon sports (carrier pigeons) [3]. The carrier pigeons are used extensively for racing, and various studies have explored the influence of genetics and dietary parameters as well as infections on race performance [4,5,6,7]. The races have a very high physical burden on racing pigeons as they are held every week for 4 consecutive months with racing distancing ranging from 100 to 1000 km [8]. During preparations for pigeon races and training, the practice of long communal transporting (sometimes for a period of 2 days) of all pigeons taking part in a certain competition is very common [9]. During the transportation stops, the practice of joint feeding and watering birds from disparate lofts is a standard procedure and can create ideal conditions for the transmission and long-distance dissemination of various pathogens, especially viruses [10]. Multiple viruses have been associated and shown to infect pigeons, including those in the families *Adenoviridae, Anelloviridae, Circoviridae*, *Herpesviridae, Paramyxoviruses*, *Parvoviridae*, *Picornaviridae and Reoviridae* [11,12,13,14,15,16,17,18].

Pigeon circovirus (PiCV, family *Circoviridae*)*,* also referred to as columbid circovirus, has a single-stranded circular DNA genome of ~2 kb that encodes two bidirectionally transcribed genes, a replication-associated protein (*rep*) and capsid protein (*cp*) genes [19,20,21]. PiCV is found globally in both feral and domesticated pigeon populations, and its prevalence can reach up to 70% depending on age and overall health status [5,22,23,24,25,26,27,28,29]. Studies have shown that pigeon circovirus can be transmitted both horizontally via the fecal-oral route and vertically [30,31,32]. Although PiCV has not been shown to directly cause any major disease in pigeons, studies have associated it with immunosuppression in younger pigeons (<4 months) [33,34].

The phylogeny of PiCV sequences does not show any clear geographic structuring [35]. However, not all PiCV variants are widely distributed. The most common PiCV variant has been detected in various European countries, United States, China, Brazil and Australia, whereas the second common variant in Europe, China, Australia and Brazil [26,27,35]. The rest of the variants are less frequent, with most identified in birds in Europe and China [26,27,35]. Perhaps the absence of geographic clustering of PiCV sequence variants could be due to the role recombination, and nucleotide substitution plays in its evolution as well as broader animal movements. Previous studies have shown a recombination hotspot within the intergenic region between *cp* and *rep* genes and near the origin of replication [27,35].

In recent years, a popular pigeon sport called “one loft races” (OLR) involving young pigeons has become popular. In this type of competition, all young birds supplied by various breeders are identically housed, fed and trained before the racing season in a single loft. This procedure usually takes 2–3 months with an aim to exclude additional factors affecting the result of the race, such as the location of the loft of origin, care and nutrition, which means that the race can be won by the best individual. However, OLR likely violates all biosecurity measures and can lead to the transmission of various pathogens. Thus, the morbidity of pigeons in OLR can be very high, and if proper veterinary care is not provided, the mortality rates can also be very high. The prevalence of PiCV in young racing pigeons is close to ~80% [30]. Taking together the sequence diversity of PiCV and the high prevalence of infections in young pigeons, it is certain that numerous PiCV variants are introduced and spread at OLRs. Furthermore, OLRs could facilitate the emergence of new recombinant PiCV variants.

In this pilot study, we investigate the recombination dynamics of PiCV in young domesticated pigeons sourced from different facilities and then housed in a common loft.

## 2. Materials and Methods

### 2.1. Ethical Statement

The research protocol was approved by the Local Ethics Committee on Animal Experimentation of the University of Warmia and Mazury in Olsztyn (resolution No 41/2019, issued 28 May 2019, valid through 1 October 2023). The researchers made every effort to minimize the suffering of birds.

### 2.2. Pigeon Lofts and Sample Collection

Eight-week-old pigeons (*n* = 10) were randomly selected and bought from three different pigeon lofts of ca. 50 birds located in Warmińsko-Mazurskie voivodeship in Poland. The lofts where the pigeons were sourced from were not connected in any way, so the passive transfer of viruses via equipment, humans or food was unlikely. In all of the pigeon lofts, PiCV was detected in the past during routine checks, but there were no clinical symptoms of any disease in the animals on the day of purchase. The young birds purchased had not been vaccinated against common viral and bacterial pathogens.

**Figure 1:**
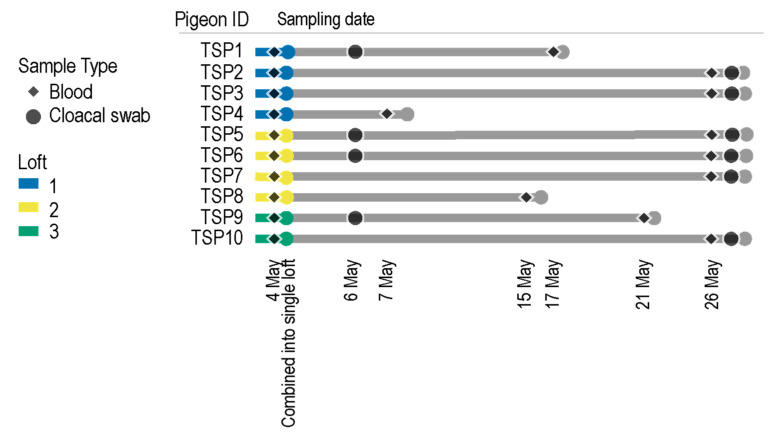
A summary of the timeline of blood and cloacal swab sampling of the pigeons (TSP1-10) for the study.

Birds TSP1-4 originated from loft 1 located in Olsztyn (racing pigeons), birds TSP5-8 originated from loft 2 located in Bartoszyce (ornamental pigeons), and birds TSP9-10 originated from the loft 3 located in Ruś (racing pigeons). Blood samples were collected on 4 May 2020 from each of these purchased pigeons. Following this, the ten pigeons (TSP1-10) were housed in a single loft. Four of the birds died during the experiment because of severe dehydration and fibrinous aerosacculitis, probably as a result of stress and mixed infection with bacteria and immunosuppressive PiCV. However, no detailed laboratory examination of deceased pigeons was performed. For the deceased pigeons, the final blood collection date was taken when they died, whereas the second date for the rest of the birds is 26 May 2020 (Figure 1). Cloacal swabs were also collected from some of the birds opportunistically to determine the viruses being shed in fecal samples (Figure 1). Cloacal swabs were not part of the main study design but proved useful in identifying PiCVs as well as a phapecoctavirus (family *Myoviridae*) [36].

### 2.3. Viral DNA Extraction and Recovery of PiCV Genomes

DNA was extracted from 10 µL of blood samples using DNeasy Blood & Tissue Kit (Qiagen, Hilden, Germany) in accordance with the manufacturer’s instructions. For cloacal swabs, viral DNA was isolated from 200 µL of UTM (Copan Diagnostics, Murrieta, CA, USA) using the High Pure viral nucleic acid kit (Roche Diagnostics, Basel, Switzerland) according to the manufacturer’s instructions.

The DNA from the individual blood and cloacal swab samples was amplified using rolling circle amplification (RCA) with TempliPhi 2000 kit (GE Healthcare, Chicago, IL, USA). Abutting primers (PICV_NF: 5’-VCG TGA CTT CAA AAC GGA AGT CAT C-3’, PICV_NR: 5’-GGM TGC TGA CCA ATC AGC AGC TT-3’) were designed in a conserved region of PiCV based on an alignment of all PiCV genome sequences available in GenBank (downloaded 14 December 2020). This primer pair was used to amplify the PiCV genomes from the RCA products using polymerase chain reaction (PCR) with Kapa HiFi DNA polymerase (Kapa Biosystems, Wilmington, MA, USA) with the following thermal cycling condition: initial denaturation 95 °C for 3 min followed by 30 cycles of 98 °C for 20 s, 55 °C for 15 s, 72 °C for 2 min and a final extension at 72 °C for 2 min followed by cooling to 4 °C for 10 min. The PCR amplicons were resolved on a 0.7% agarose gel, and ~2 kb amplicons were excised from the agarose gel and purified. The purified amplicons were ligated into the pJET 1.2 vector (Thermo Fisher Scientific, Waltham, MA, USA), and the recombinant plasmids were transformed into *Escherichia coli* XL blue competent cells. Ten clones were selected from the blood, and 5 from each swab positive sample for Sanger sequencing of the PiCV-cloned genomes. The recombinant plasmids were purified using Fast DNA-spin Plasmid DNA Purification Kit (iNtRON Biotechnology, Seongnam, Gyeonggi, Korea) and Sanger sequenced at Macrogen Inc. (Seoul, Korea) by primer walking. The sequences were assembled and annotated using Geneious v11.0.3. 178 (Biomatters Ltd., Auckland New Zealand). Poorly sequenced clones were not included in downstream analysis. The assembled sequences from high-quality Sanger-sequenced clones were deposited in GenBank (accession # are listed in Table 1).

### 2.4. Bioinformatic Analyses of PiCV Genome Sequences

Complete PiCV genomes determined in this study together with those available in GenBank (downloaded on 15 February 2021) and beak and feather disease virus sequence as an outgroup were aligned using MUSCLE [37]. This alignment was used to infer a Neighbor-Joining phylogenetic tree using Mega X [38] with the Jukes-Cantor substitution model and 1000 bootstrap replicates. Branches with <60% bootstrap support were collapsed using TreeGraph2 [39]. Genome-wide pairwise identities were determined using SDT 1.2 [40].

Recombination analysis was performed using RDP5 [41] with default settings, and only recombination events that were detected by more than three methods with a *p*-value < 0.05 were accepted as credible.

The alignment of the sequences, with recombinant regions removed, was used to infer a Maximum Likelihood phylogenetic tree using PhyML [42] with K2 + G + I nucleotide substitution model and 1000 bootstrap replicates. Branches with <60% bootstrap support were collapsed using TreeGraph2 [39].

## 3. Results and Discussion

### 3.1. Characterization of PiCV Genotypes

**Figure 2:**
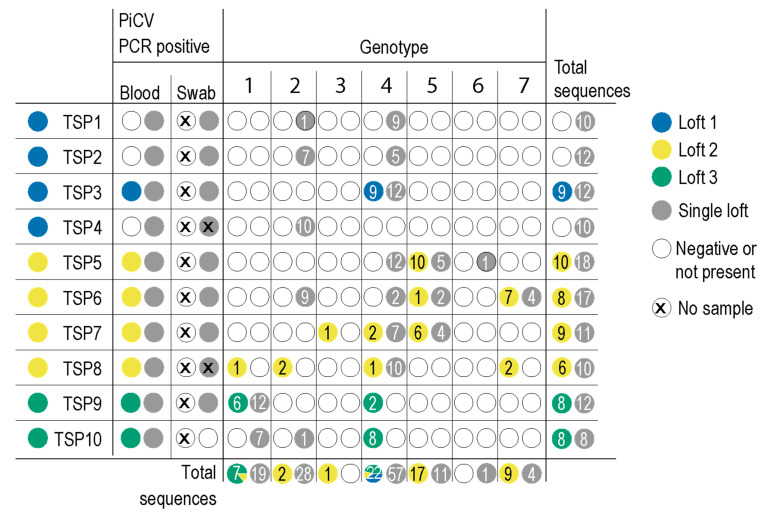
A summary of the PiCV genotypes identified in the study in either the blood or cloacal swab samples. The pigeons were sourced from three separate lofts (loft 1–3) and then house in a single loft. Blood samples were taken when sourced, and thereafter, blood and cloacal swabs were taken at different time points as outlined in Figure 1. Genotypes identified in animals in each of the three lofts and also once they are housed in a single loft are color-coded, and the number in the circles indicates the number of genomes determined that are assigned to the genotype.

Ten birds were purchased from three different lofts, and blood samples were collected from each animal. Thereafter, they were all housed in a single loft for 22 days, and blood and cloacal swab samples were collected as summarized in Figure 1 and Figure 2. A total of 58 genome sequences were determined from seven pigeons before they were housed together in a loft and 120 thereafter (Table 1, Figure 2). Pigeons TSP1, TSP2 and TSP4, were identified as PiCV negative prior to grouping them in one loft.

The 178 PiCV genome sequences from this study were analyzed together with all PiCV full genomes available in GenBank (*n* = 127). Pairwise identity analyses showed the all PiCVs (those available in GenBank and from this study) share >83% genome-wide identity. Based on the genome-wide pairwise identities, we identified seven genotypes for PiCV genome sequences based on the criteria we set for the purpose of this study, which was that viral sequences that share >98% genome-wide identity are part of the same genotype (Figure 2). Additionally, 95 genotypes could represent all other PiCV sequences publicly available (Supplementary Data S1).

PiCV genotypes 1, 2, 3, 4, 5 and 7 were present in the pigeons from the three lofts (Table 1, Figure 2). In the case of TSP3, from loft 1, only genotype 4 was initially identified. Once the pigeons from loft 1 (TSP1-4) were moved to a single loft, PiCV genotypes 2 and 4 were detected in TSP1 and TSP2, and genotype 2 in TSP4, suggesting that these animals became infected subsequent to being moved to a single loft. PiCV genotypes 1, 2, 3, 4, 5 and 7 were identified in TSP5-8 from loft 2; however, after moving them to a single loft, we identified genotypes 2, 4, 5, 6 and 7 and not genotypes 1, 3 and 5. It is interesting to note that animal TSP6 was found harboring four PiCV genotypes (Figure 2). PiCV genotype 3 was detected in animal TSP7 initially, and this is the only animal to have this genotype. PiCV genotype 6 was identified only in animal TSP5 once all of the birds were housed in a single loft. In the two animals from loft 3, PiCV genotypes 1 and 4 were identified. Once moved to a single loft, there is a shift in genotypes to 1 and 2 (Figure 2).

In general, genotype 4 was highly prevalent, making up 37.9% (22/58) of the PiCV sequences in five pigeons in the three lofts prior to being housed in a single loft. Once all the animals were housed in the same loft, this genotype was found in seven pigeons, which accounts for 47.5% (57/120) (Figure 2).

### 3.2. Phylogenetic Analysis of PiCV Genomes

**Figure 3:**
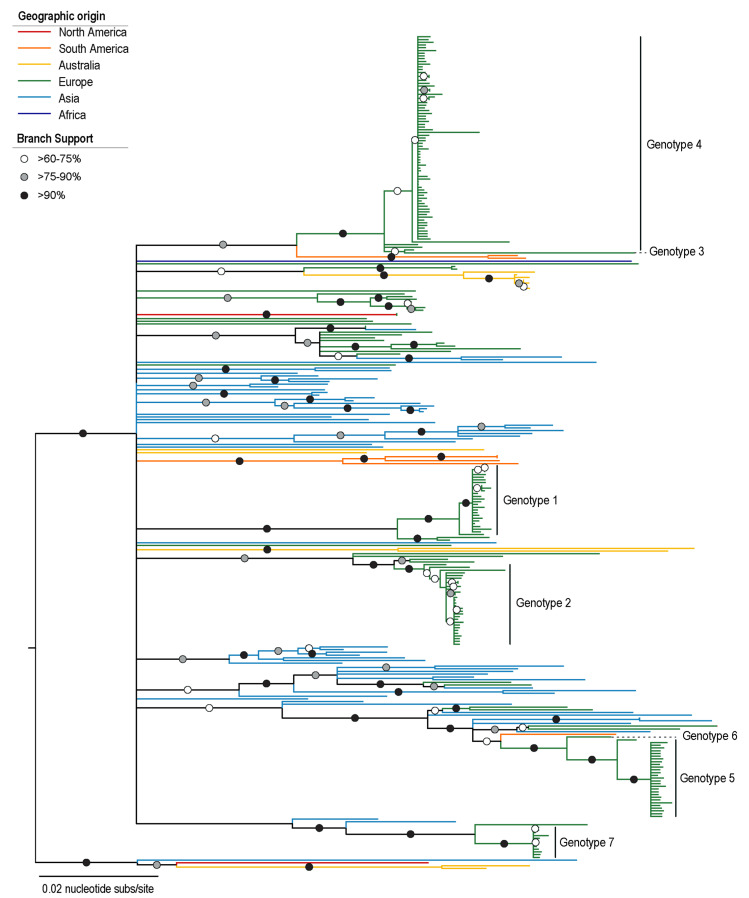
Neighbor-Joining phylogenetic tree of the PiCV genome sequences from this study together with those available in GenBank. Branches are color-coded based on the geographical sampling of the PiCV. The genotypes 1–7 represent sequences from this study.

PiCV genomes available in GenBank (download 15 February 2021) are those identified in pigeons from Australia (*n* = 13), Belgium (*n* = 6), Brazil (*n* = 7), China (*n* = 63), France (*n* = 1), Japan (*n* = 1), Nigeria (*n* = 1), Poland (*n* = 30), United Kingdom (*n* = 2) and United States of America (*n* = 3). Global phylogenetic analysis of the PiCV recovered from 10 pigeons in this study (*n* = 178) together with those PiCV genomes available in GenBank was undertaken (Figure 3). The phylogenetic analysis coupled with genotype assignment shows that PiCV sequences from this study are distributed within seven distinct clades according to our genotype designation based on >98% nucleotide pairwise identity threshold. Genotypes 3, 4, 5 and 6 form clades that are most closely related to PiCV sequences identified in a pigeon from Brazil and those from China and Poland. Genotypes 1 and 2 sequences form clades with sequences most closely related to PiCVs from birds sampled in Poland. Lastly, genotype 7 sequences cluster with two PiCV sequences from China. The PiCV genotypes identified in this study are distributed throughout the phylogenetic tree, clearly highlighting diverse PiCVs circulating in at least three of the lofts from which the pigeons were acquired. Furthermore, we detected multiple genotypes within a single animal (Figure 2).

### 3.3. Recombination Analysis

**Figure 4:**
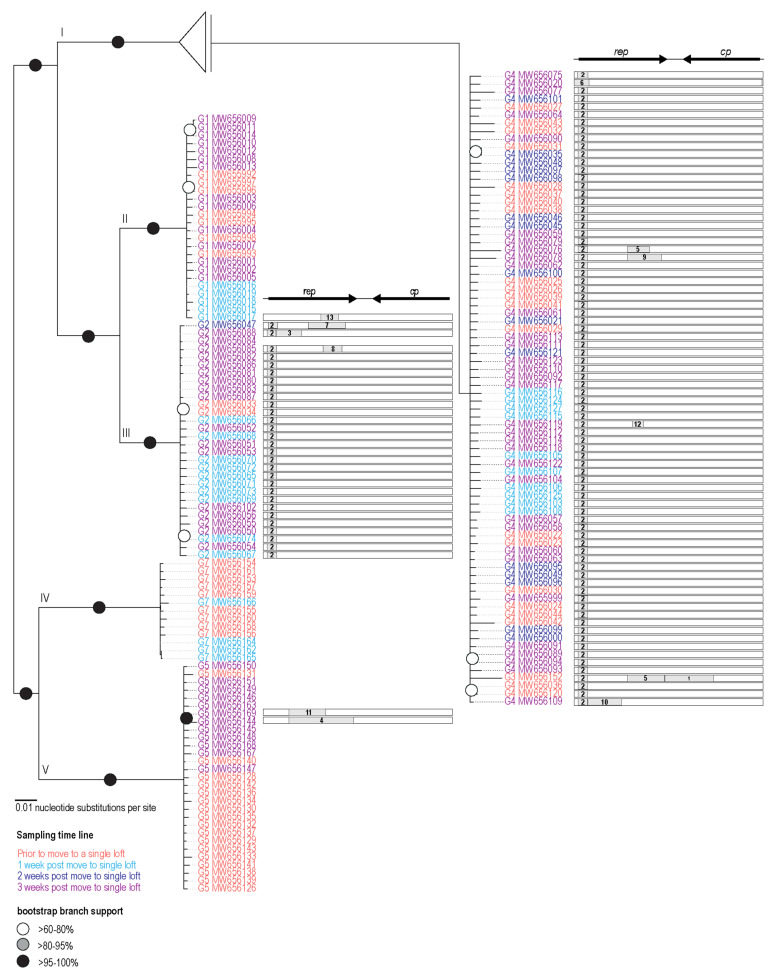
Maximum Likelihood phylogenetic tree of PiCV sequences recovered from study with recombinant regions removed. Recombinant regions identified in the genomes are shown next to the accession numbers. The five main clades are labeled in roman numerals (I–V). Genotypes (G1–G7) are labeled next to all the accession numbers.

**Figure 5:**
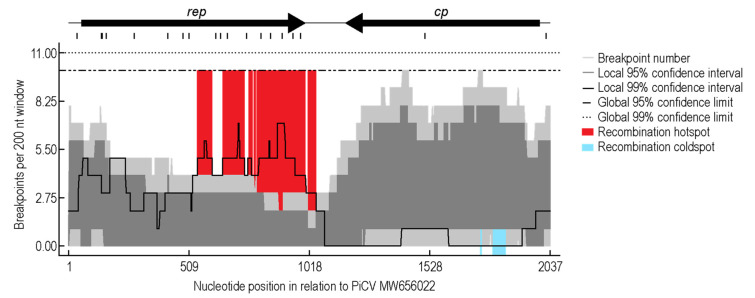
Recombination break point analysis for PiCV genomes from this study. Hot spots and cold spots are highlighted. Genome organization based on PiCV sequence MW656022 as a representative.

To investigate PiCV recombination dynamics, a pilot simulation of “housing together” naturally infected and non-infected animals originated from different maternal lofts was undertaken. The recombination analysis of the PiCV sequences from this study revealed 13 detectable recombination events. The recombination event detected in a large portion of the sequences was event 2, occurring in 109 genomes (Figure 4), and this recombinant region spans a ~100 nt region located at the 5’ portion of *rep*. Twelve of the recombination events detected are within the *rep* gene (Figure 4). We identified a recombination hotspot spanning the 3′ region of *rep* and the intergenic region, whereas a cold spot was identified near the 5′ region of the *cp* (Figure 5). These results differ from the previous hotspot detected in PiCV [35], which shows hotspots towards the 3′ end of the *rep* and *cp*, as well as the intergenic region. The differences in recombination patterns could be due to the variation in samples, i.e., global datasets with “circulating” recombinants as opposed to the ones in this study that address recombination in a short time frame within a small set of animals in a loft. The cold spot located in the *cp,* along with low amounts of recombination events, shows a higher region of conservation and importance of the *cp* likely for transmission and infectivity. Phylogenetic analysis of the genomes without recombination shows five clades present in the population (Figure 4) in contrast to the seven genotypes present in the Neighbor-Joining phylogenetic analysis (Figure 3).

There is no evidence of recombination in sequences present in Clade II and IV (Figure 4). Clade IV (Figure 4) is mainly composed of sequences determined from animals prior to being in a single loft and those after being in a single loft for a week. PiCV sequence MW656152 from animal TSP7 had three recombinant regions accounting for 42% of the genome, but this was from a sample taken from the animal while in maternal loft 2, and this represents PiCV recombination within the loft of origin (Figure 4; Table 1 and Table 2). PiCV sequence MW656144 from TSP5 has a recombinant region, accounting for 34% of the genome. Both PiCV sequences MW656152 and MW656144 are singletons forming their own genotypes 3 and 6, respectively (Figure 3, Supplementary Data S1).

Only three recombination events were identified in sequences derived from the animals while in the three separate lofts of origin, whereas ten recombination events were detected in sequences derived from animals once they were housed in the same loft.

## 4. Concluding Remarks

In this study, we undertook a pilot experiment to simulate the viral dynamics at play when infected and non-infected pigeons are acquired from different sources and then housed together in a single loft. We show that genotypes of PiCV spread to non-infected birds and also to already infected birds, either resulting in co-infection or displacement of the previous genotype, e.g., the genotype 4 in animal TSP7 that appears to have “out competed” genotypes 1, 2 and 7 (Figure 2). We also demonstrate that recombination plays a role in virus evolution.

This study demonstrates the PiCV spreads in animals within a loft in a short time frame, in which the animals can harbor multiple genotypes and dominant genotypes possibly outcompete previous variants. Pigeon circovirus is known as an immunosuppressive factor in pigeons. A previous study performed on 30 PiCV full genomes recovered from pigeons of various breeds and different health statuses did not find any correlation between genotype, sequence mutation of circovirus strain and health status of its host [18]. There is also no information concerning a similar correlation between the certain/dominant genotype and immunosuppression of the pigeons. However, this phenomenon cannot be excluded at this stage.

## Figures and Tables

**Table 1:** The collection date and sampling data of PiCV genome sequences from the study. PiCV sequence accession numbers and the number of isolates from samples are listed.

Pigeon	Date	Blood	Cloacal Swab	Accession #s
TSP1	4 May 2020	x		negative
	13 May 2020	x		MW656045, MW656046, MW656047, MW656048, MW656049
	6 May 2020		x	MW656103, MW656106, MW656107, MW656108, MW656125
TSP2	4 May 2020	x		negative
	26 May 2020	x		MW656050, MW656051, MW656052, MW656053, MW656054, MW656055, MW656056
	26 May 2020		x	MW656109, MW656110, MW656111, MW656104, MW656123
TSP3	4 May 2020	x		MW656022, MW656023, MW656024, MW656025, MW656026, MW656027, MW656028, MW656029, MW656030
	26 May 2020	x		MW656057, MW656058, MW656059, MW656060, MW656061, MW656062, MW656063, MW656064
	26 May 2020		x	MW656112, MW656113, MW656114, MW656122
TSP4	4 May 2020	x		negative
	7 May 2020	x		MW656065, MW656066, MW656067, MW656068, MW656069, MW656070, MW656071, MW656072, MW656073, MW656074
TSP5	04 May 2020	x		MW656128, MW656129, MW656130, MW656131, MW656132, MW656133, MW656134, MW656126, MW656135, MW656136
	6 May 2020		x	MW656105, MW656115, MW656116, MW656124, MW656127
	26 May 2020	x		MW655999, MW656079, MW656075, MW656076, MW656077, MW656078, MW656020, MW656144
	26 May 2020		x	MW656145, MW656149, MW656150, MW656151, MW656168
TSP6	4 May 2020	x		MW656153, MW656154, MW656159, MW656137, MW656155, MW656156, MW656157, MW656158
	6 May 2020		x	MW656162, MW656164, MW656165, MW656166
	26 May 2020	x		MW656080, MW656081, MW656082, MW656083, MW656084, MW656085, MW656086, MW656087, MW656088
	26 May 2020		x	MW656118, MW656119, MW656146, MW656169
TSP7	4 May 2020	x		MW656138, MW656143, MW656152, MW656139, MW656140, MW656031, MW656141, MW656120, MW656142
	26 May 2020	x		MW656089, MW656090, MW656091, MW656092, MW656093, MW656094
	26 May 2020		x	MW656147, MW656148, MW656117, MW656163, MW656167
TSP8	4 May 2020	x		MW655992, MW656032, MW656033, MW656034, MW656160, MW656161,
	15 May 2020	x		MW656000, MW656021, MW656095, MW656096, MW656097, MW656098, MW656099, MW656100, MW656101, MW656121
TSP9	4 May 2020	x		MW655993, MW655994, MW655995, MW655996, MW655997, MW656036, MW655998, MW656035
	06 May 2020		x	MW656015, MW656016, MW656017, MW656018, MW656019
	21 May 2020	x		MW656001, MW656002, MW656003, MW656004, MW656005, MW656006, MW656007
TSP10	4 May 2020	x		MW656037, MW656038, MW656039, MW656040, MW656041, MW656042, MW656043, MW656044
	26 May 2020	x		MW656008, MW656009, MW656010, MW656011, MW656102, MW656012, MW656013, MW656014

x = sampled.

**Table 2:** A summary of the recombination events detected in the genomes of PiCV from this study. The methods used to detect recombination are RDP (R), GENCONV (G), BOOTSCAN (B), MAXCHI (M), CHIMERA (C), SISCAN (S) and 3SEQ (T). For each recombination event, the method with the highest *p*-value is in bold font and underlined. [P] denotes partial evidence of recombination.

Event #		Recombinant Region		Minor Parental Sequence(s)	Major Parental Sequence(s)	Method	*p*-Value
	Recombinant Sequence(s)	Begin	End				
1	Genotype 3	983	1512	Genotype 6 (all) Genotype 5 (MW656131, MW65613, MW656150, MW656169)	Genotype 4 (all)	GBMCS**T**	8.88 × 10^−47^
2	Genotype 4 (all) Genotype 2 (all) Genotype 3	38	140	Genotype 5 (all)	Genotype 1 (all)	**G**BMCS	6.04 × 10^−20^
3	Genotype 2 (MW656088)	142	418	Genotype 7 (all) Genotype 5 (MW656159)	Genotype 2 (expect MW656088)	**G**MCST	2.77 × 10^−17^
4	Genotype 6	275	983	Genotype 4 (MW656020)	Genotype 5 (all)	**G**MCST	4.69 × 10^−16^
5	Genotype 4 (MW656076) Genotype 3 (MW656152)[P]	577 *	816	Genotype 5 (all)	Genotype 4 (MW656020, MW656093, MW656106, MW656109, MW656120)	**G**MCST	1.52 × 10^−13^
6	Genotype 4 (MW656020)	2018	158	Genotype 1 (all)	Genotype 2 (all)	**G**MCST	2.46 × 10^−13^
6	Genotype 4 (MW656020)	2018	158	Genotype 1 (all)	Genotype 2 (all)	**G**MCST	2.46 × 10^−13^
7	Genotype 2 (MW656047)	484	904	Genotype 4 (MW656020)	Genotype 2 (MW656085)	G**B**MCST	2.59 × 10^−13^
8	Genotype 2 (MW656085)	644	856	Genotype 7 (all)	Genotype 2 (except MW656085)	**G**BMCST	4.99 × 10^−12^
9	Genotype 4 (MW656078)	577*	948	Genotype 1 (all)	Genotype 4 (except MW656078)	**G**BMCST	5.69 × 10^−11^
10	Genotype 4 (MW656109)	142*	508	Genotype 5 (all)	Genotype 4 (MW656093, MW656119, MW656020) Genotype 3	GBMCS**T**	8.88 × 10^−22^
11	Genotype 5 (MW656169)	277	676	Genotype 4 (all)	Genotype 5 (except MW656169)	G**B**MCST	3.20 × 10^−17^
12	Genotype 4 (MW656119)	624	752	Genotype 5 (all)	Genotype 4 (except MW656119) Genotype 2 (all)	G**B**T	2.32 × 10^−9^
13	Genotype 1 (MW656017)	621	813	Genotype 5 (all)	Genotype 1 (except MW656017)	G**B**T	1.29 × 10^−8^

## Data Availability

Sequence determined as part of this study have been deposited in GenBank under accession #s MW655992 - MW656169.

## Supplementary Materials

The following are available online at https://www.mdpi.com/article/10.3390/v13060964/s1, Supplementary Data S1: Nucleotide pairwise identity analysis of all PiCV sequences (including those from this study).
